# Supplementary material for: An open-source framework for large-scale, flexible evaluation of biomedical text mining systems
Source: J Biomed Discov Collab. 2008 Jan 29;3:1. doi: 10.1186/1747-5333-3-1 (PMC2276192; doi:10.1186/1747-5333-3-1)
Supplement: Additional file 1 — Evaluation platform source code. The additional file contains the software evaluation framework discussed in this paper. The most current release can be downloaded from SourceForge.net [1]. See the accompanying README file for instructions on installation and use. Also included in the distribution is a collection of UIMA wrappers for some commonly used BioNLP tools and annotated corpora. [file 1747-5333-3-1-S1.zip › bionlp_uima_repository-1.0.1/docs/api/src-html/edu/uchsc/ccp/uima/ae/util/converter/AnnotationSetOverrider_AE.html]

```
001    package edu.uchsc.ccp.uima.ae.util.converter;
002    
003    import java.util.Arrays;
004    import java.util.HashSet;
005    import java.util.Iterator;
006    import java.util.Set;
007    
008    import org.apache.uima.analysis_engine.ResultSpecification;
009    import org.apache.uima.analysis_engine.annotator.AnnotatorConfigurationException;
010    import org.apache.uima.analysis_engine.annotator.AnnotatorContext;
011    import org.apache.uima.analysis_engine.annotator.AnnotatorContextException;
012    import org.apache.uima.analysis_engine.annotator.AnnotatorInitializationException;
013    import org.apache.uima.analysis_engine.annotator.AnnotatorProcessException;
014    import org.apache.uima.analysis_engine.annotator.JTextAnnotator_ImplBase;
015    import org.apache.uima.jcas.JCas;
016    import org.apache.uima.jcas.cas.FSArray;
017    
018    import edu.uchsc.ccp.uima.annotation.CCPAnnotationSet;
019    import edu.uchsc.ccp.uima.annotation.CCPTextAnnotation;
020    /**
021     * Changes annotation set references unless, the annotation has a set in the annotation sets to ignore 
022     * @author williamb
023     *
024     */
025    public class AnnotationSetOverrider_AE extends JTextAnnotator_ImplBase {
026    
027        public static final String PARAM_SETID = "SetID";
028        public static final String PARAM_SETNAME = "SetName";
029        public static final String PARAM_SETDESCRIPTION = "SetDescription";
030        public final String PARAM_ANNOTATION_SETS_TO_IGNORE = "AnnotationSetsToIgnore";
031        protected Set<Integer> annotationSetsToIgnore;
032        private int setID = -1;
033        private String setName = null;
034        private String setDescription = null;
035        
036        @Override
037        public void initialize(AnnotatorContext ac) throws AnnotatorInitializationException, AnnotatorConfigurationException {
038            /* read in input parameters */
039            try {
040                setID = ((Integer) ac.getConfigParameterValue(PARAM_SETID)).intValue();
041                setName = (String) ac.getConfigParameterValue(PARAM_SETNAME);
042                setDescription = (String) ac.getConfigParameterValue(PARAM_SETDESCRIPTION);
043                annotationSetsToIgnore = new HashSet<Integer>(Arrays.asList((Integer[]) ac
044                        .getConfigParameterValue(PARAM_ANNOTATION_SETS_TO_IGNORE)));
045            } catch (AnnotatorContextException e) {
046                e.printStackTrace();
047            }
048            super.initialize(ac);
049        }
050        
051        
052        /**
053         * cycle through all annotations and set the annotation set
054         */
055        public void process(JCas jcas, ResultSpecification arg1) throws AnnotatorProcessException {
056    
057            /* create an annotation set list */
058            CCPAnnotationSet ccpAnnotationSet = new CCPAnnotationSet(jcas);
059            ccpAnnotationSet.setAnnotationSetID(setID);
060            ccpAnnotationSet.setAnnotationSetName(setName);
061            ccpAnnotationSet
062                    .setAnnotationSetDescription(setDescription);
063            FSArray annotationSets = new FSArray(jcas, 1);
064            annotationSets.set(0, ccpAnnotationSet);
065    
066            Iterator annotIter = jcas.getJFSIndexRepository().getAnnotationIndex(CCPTextAnnotation.type).iterator();
067    
068            while (annotIter.hasNext()) {
069                Object possibleAnnot = annotIter.next();
070                if (possibleAnnot instanceof CCPTextAnnotation) {
071                    CCPTextAnnotation ccpTA = (CCPTextAnnotation) possibleAnnot;
072                    boolean ignore = checkForIgnoreBasedOnAnnotationSet(ccpTA);
073    
074                    if (!ignore) {
075                        ccpTA.setAnnotationSets(annotationSets);
076                    }
077                } else {
078                    System.err.println("WARNING -- AnnotationSetValidator_AE: CCPTextAnnotation expected but instead got "
079                            + possibleAnnot.getClass().getName());
080                }
081            }
082    
083        }
084    
085        protected boolean checkForIgnoreBasedOnAnnotationSet(CCPTextAnnotation ccpTA) {
086            boolean ignore = false;
087            FSArray annotationSets = ccpTA.getAnnotationSets();
088            for (int i = 0; i < annotationSets.size(); i++) {
089                CCPAnnotationSet aSet = (CCPAnnotationSet) annotationSets.get(i);
090                if (annotationSetsToIgnore.contains(aSet.getAnnotationSetID())) {
091                    ignore = true;
092                    break;
093                }
094            }
095            return ignore;
096        }
097    
098    }
```
